# Single-cell protein production from CO_2_ and electricity with a recirculating anaerobic-aerobic bioprocess

**DOI:** 10.1016/j.ese.2025.100525

**Published:** 2025-01-10

**Authors:** Zeyan Pan, Yuhan Guo, Weihe Rong, Sheng Wang, Kai Cui, Wenfang Cai, Zhihui Shi, Xiaona Hu, Guokun Wang, Kun Guo

**Affiliations:** a School of Chemical Engineering and Technology, Xi'an Jiaotong University, Xi'an, 710049, China; b Key Laboratory of Engineering Biology for Low-carbon Manufacturing, Tianjin Institute of Industrial Biotechnology, Chinese Academy of Sciences, Tianjin, 300308, China; c Shanghai Zelixir Biotech Company Ltd., Shanghai, 200030, China; d School of Ecology and Environment, Zhengzhou University, Zhengzhou, 450000, China; e Xi’an Key Laboratory of C1 Compound Bioconversion Technology, Xi’an Jiaotong University, Xi’an, 710049, China

**Keywords:** Microbial electrosynthesis, Microbial acetate upgrading, Single-cell protein, CO_2_ utilization, Acetate

## Abstract

Microbial electrosynthesis (MES) represents a promising approach for converting CO_2_ into organic chemicals. However, its industrial application is hindered by low-value products, such as acetate and methane, and insufficient productivity. To address these limitations, coupling acetate production via MES with microbial upgrading to higher-value compounds offers a viable solution. Here we show an integrated reactor that recirculates a cell-free medium between an MES reactor hosting anaerobic homoacetogens (*Acetobacterium*) and a continuously stirred tank bioreactor hosting aerobic acetate-utilizing bacteria (*Alcaligenes*) for efficient single-cell protein (SCP) production from CO₂ and electricity. The reactor achieved a maximum cell dry weight (CDW) of 17.4 g L^−1^, with an average production rate of 1.5 g L^−1^ d^−1^. The protein content of the biomass reached 74% of the dry weight. Moreover, the integrated design significantly reduced wastewater generation, mitigated product inhibition, and enhanced SCP production. These results demonstrate the potential of this integrated reactor for the efficient and sustainable production of high-value bioproducts from CO_2_ and electricity using acetate as a key intermediate.

## Highlights

• A hybrid bioreactor integrating anaerobic and aerobic processes is developed for converting electricity into SCP.
• The system facilitates collaboration between anaerobic *Acetobacterium* and aerobic *Alcaligenes.*
• Efficient SCP production from CO_2_ is achieved using acetate as the intermediate metabolite.
• The reactor design mitigates product inhibition, reduces base consumption, and minimizes wastewater generation.

## Graphical abstract

**Figure:**
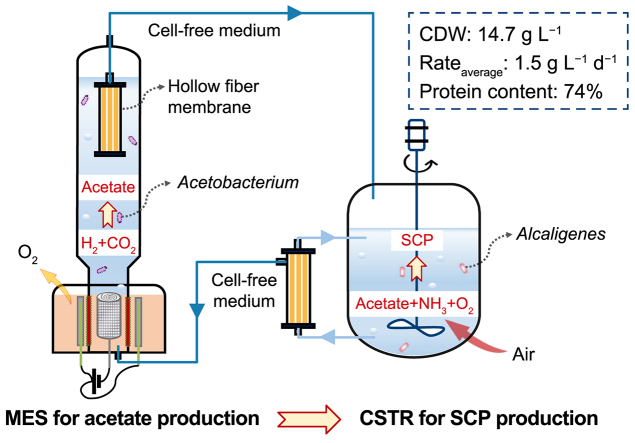

## 1 Introduction

Climate change caused by increasing carbon dioxide (CO_2_) emissions has adversely affected the global economy and environmental stability [1]. Hence, the artificial recycling of CO_2_ into valuable products is an important approach to addressing environmental issues and is, in fact, key to the establishment of a climate-friendly circular carbon economy [2]. In this regard, interest in microbial electrosynthesis (MES) is growing in the environment and energy fields as an emerging electrochemical technology for microbial conversion of CO_2_ into biochemicals [[3], [4], [5], [6]]. MES has distinct advantages over traditional electrochemical CO_2_ reduction methods, including enhanced product selectivity, improved energy efficiency, and long-term stability [7,8]. Unlike direct electron transfer, hydrogen mediated (H_2_-mediated) MES allows for higher cathode current density without requiring a biofilm. However, its productivity is low, as its primary product is acetate, which has a low added value and high separation cost. Therefore, upgrading this acetate into a broad spectrum of higher-value products is essential for the practical application of MES.

To produce higher-value MES products by increasing acetate's product value and decreasing its separation cost, two-stage processes have been developed that integrate MES into acetate-utilizing processes. For example, Molitor et al. [9] reported that acetate produced by anaerobic acetogenic bacteria could grow single-cell protein (SCP) in yeast under aerobic conditions. Similarly, Yadav et al. [10] demonstrated a two-stage process in which, in the first stage, MES converts CO_2_ into acetate; and in the second stage, the acetate provides the carbon needed by *Saccharomyces cerevisiae* to produce sclareol, β-carotene, and yeast biomass. These two-stage processes are quite promising. However, in their reported implementations, the medium was not recirculated (i.e., the reactor was running in one-way mode), which resulted in substantial wastewater generation and nutrient wastage. Moreover, as the acetate was not simultaneously removed from the first MES reactor, the product inhibition in MES could not be alleviated. Therefore, a more advanced two-stage process is needed to enhance productivity and efficiency.

To recirculate the medium between the MES and the second reactor, the microorganisms in the two reactors should have similar optimal pH ranges and medium requirements. Hence, it is challenging to couple bacteria and yeast into a circulation system. While the pH and medium requirements of *Alcaligenes*, a bacterium that can utilize acetate to produce SCP, are similar to those of the homo-acetogens in MES, those of yeast are not [11]. More importantly, in MES, homo-acetogens tend to lower the pH of the medium due to acetate production, whereas *Alcaligenes* raise the pH during their growth with acetate. Therefore, we hypothesize that coupling *Alcaligenes* and homo-acetogens into a circulation system could theoretically reduce wastewater generation, alleviate product inhibition, and reduce acid/base consumption. However, the implementation of such a two-stage process with medium recirculation has yet to be documented.

**Fig. 1:**
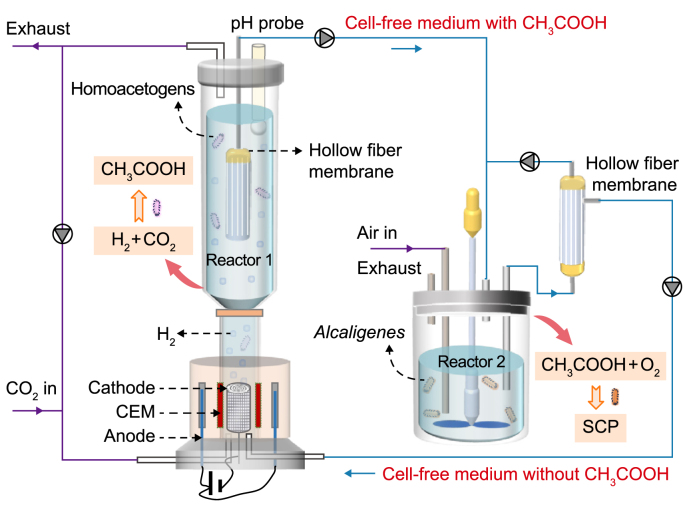
Schematic of single**-**cell protein (SCP) production process using microbial conversion. In Reactor 1, CO_2_ is converted to acetate by microbial electrosynthesis. In Reactor 2, this acetate is fermented to SCP by acetate-utilizing bacteria. Two reactors were connected by hollow fiber membrane filters for circulated operation. CEM: cation exchange membrane.

To test our hypothesis, we developed a two-stage process that coupled an electrolytic bubble (electro-bubble) column MES reactor (Reactor 1) with a continuously stirred tank bioreactor (Reactor 2) to produce SCP from CO_2_ and electricity (Fig. 1). Reactor 1 produced acetate from CO_2_ and electricity using anaerobic homo-acetogens, and Reactor 2 upgraded the acetate into SCP using aerobic *Alcaligenes*. Both reactors were equipped with hollow fiber membranes that enabled the continuous circulation of a cell-free medium between them. Our results show that the coupled system with medium recirculation effectively reduced the wastewater generation, alleviated the product inhibition, and enhanced the SCP production. Thus, the reactor setup holds great potential for efficient and continuous SCP production from CO_2_ and electricity, with acetate serving as the intermediate metabolite.

## 2 Materials and methods

### 2.1 Medium and inoculum

In this study, Reactor 1 utilized a synthetic medium with the following components: 3 g L^−1^ KH_2_PO_4_, 6 g L^−1^ Na_2_HPO_4_, 6.1 g L^−1^ NH_4_Cl, 0.1 g L^−1^ MgSO_4_·7H_2_O, 0.5 g L^−1^ NaCl, 14.6 mg L^−1^ CaCl_2_, 1 mL L^−1^ vitamin solution, and 1 mL L^−1^ trace element solution. The detailed compositions of the trace element and vitamin solutions are shown in Table S1 (Supplementary Material). Additionally, to inhibit methanogens, 15 mM sodium 2-bromoethanesulfonate was included. The inoculum utilized in Reactor 1 was obtained from the effluent of a prior MES reactor in a laboratory [12], wherein *Acetobacterium* dominated the microbial community.

The same medium was utilized in Reactor 2. A seed culture of acetate-utilizing bacteria was cultivated in a 250 mL beaker flask filled with 100 mL of the medium. After the bacterial culture was agitated overnight in a rotary shaker at 30 °C and 300 rpm, 5 mL of such culture was inoculated into another 250 mL beaker flask filled with 100 mL of the same medium. After this bacterial culture was cultivated for 12 h, it was introduced into Reactor 2.

### 2.2 Operation of the anaerobic electro-bubble column bioreactor

An electro-bubble column bioreactor with a 6 L working volume was used to grow anaerobic homo-acetogens. The anode consisted of a titanium mesh coated with IrO_2_, with a 9 cm diameter and a 10 cm height. The cathode was constructed from an 8 × 26 cm steel mesh (0.5 mm thick, 304 L), which was fashioned into a spiral configuration to promote uniform distribution of hydrogen gas bubbles. After adding the medium to the reactor, a 1 A current was constantly applied via a direct current (DC) power supply (MS-3010DS, Korad Technology Co., Ltd., Dongguan, China) to produce H_2_. Pure CO_2_ was passed into the catholyte at a 4 mL min^−1^ flow rate using a mass flow controller (D07-7, Sevenstar Flow Co., Ltd., Beijing, China). Then, H_2_ and CO_2_ were converted into acetate via anaerobic homo-acetogens. The applied current was gradually increased based on utilizing H_2_ and CO_2_, as shown in Table S2 and Fig. S3 (Supplementary Materials). A continuous gas supply was maintained, and unused gas was recycled at a 200 L h^−1^ flow rate. The temperature in the reactor was kept constant at 30 ± 0.5 °C using a silicone heating pad (B/GXJ, Songdao Heating Sensor Co., Ltd., Shanghai, China), while the pH of the catholyte was regulated at 7 by adding 5 M NaOH with a pH controller (pH61J, Tianze Biotechnology Co., Ltd., Guangzhou, China). A dissolved oxygen (DO) probe was installed on the reactor lid to detect the DO level, and the results are shown in Fig. S1 (Supplementary Material). The electro-bubble column bioreactor was inoculated with 100 mL inoculum containing a *Acetobacterium*-dominated homo-acetogen community from the MES reactor for acetate production [12]. Additionally, the reactor was equipped with a hollow fiber membrane (Rongxin Environmental Technology Co., Ltd., Zhongshan, China) to intercept bacterial cells and filter the effluent. The flow rate of the effluent in circulated mode was 7.2 mL min^−1^. To verify the reproducibility of this study, when the uptake efficiency of H_2_ and CO_2_ dropped sharply, 5.8 L of catholyte was replaced with a fresh culture medium to initiate another experiment. Concurrently, Reactor 2 was refilled with the fresh culture medium and reinoculated.

### 2.3 Operation of the continuous stirred tank aerobic bioreactor

Acetate-utilizing bacteria were cultivated in a 5 L stirred tank bioreactor (Intelli-Ferm Q, Parallel-Bioreactor Inc., Shanghai, China) with a 1.5 L working volume. Air was continuously supplied to the reactor via an air compressor. The pH and DO levels were set at 7.0 and 20%, respectively, maintained with cascade control, and closely monitored using a pH probe and a DO probe. A phosphoric acid solution was used to control the pH. A hollow fiber membrane module (Aideal Biotech Inc., Zhuhai, China) with a 0.2 μm pore rating, attached to two peristaltic pumps, was continuously employed during the reactor operation to recycle cells and eliminate excess volume introduced through acetate feeding.

### 2.4 Integrated bioprocess system

The process (Fig. 1) showcases the integration of the electro-bubble column bioreactor, used for homo-acetogens, and the stirred tank bioreactor, used for acetate-utilizing bacteria. CO_2_ was fed to the electro-bubble column and converted into acetate, which, in turn, was fed to the stirred tank reactor and converted into SCP. During the startup period of two days, the electro-bubble column bioreactor operated independently. After the activation phase, the flow of liquid media was started, and the bioreactor housing homo-acetogens was transitioned into circulation mode. This study continuously transferred the acetate-containing medium from the electro-bubble column reactor to the stirred tank bioreactor via a peristaltic pump (BT 100-2J, LongerPump Inc., Baoding, China) at a 7.2 mL min^−1^ flow rate. The medium was simultaneously inoculated with acetate-utilizing bacteria in the stirred tank bioreactor and maintained in circulation mode. To consistently maintain the working volume and effectively retain cells within the bioreactor, the solution carrying bacterial cells from the stirred tank bioreactor was circulated through a hollow fiber membrane filter module. The resulting clear effluent was sent back to the electro-bubble column reactor with a 7.2 mL min^−1^ flow rate, while the bacterial cells returned to the stirred tank bioreactor.

### 2.5 Analytical methods

To assess cell growth, substrate consumption, and product formation, daily samples were collected from Reactor 1 and Reactor 2. The off-gas in Reactor 1 was collected using the water displacement method to measure the volume, and the gas composition was analyzed using a gas chromatograph (7890B, Agilent Technologies Inc., Santa Clara, USA). The acetate analysis was performed using an additional gas chromatograph (GC-2010 Pro, Shimadzu Inc., Kyoto, Japan). Detailed methods can be found in our previous publication [13]. Bacterial growth was monitored by measuring the optical density at 600 nm (OD_600_) (Biotek Synergy H1, BioTek Instruments Inc., Vermont, USA) and the weight of the dried biomass. A 40 mL broth sample was collected to determine the cell dry weight (CDW). This sample was centrifuged at 10,000 rpm for 10 min, after which the supernatant was removed. The remaining biomass was washed three times with deionized water to eliminate inorganic salts, and then, transferred to a preweighed 40 mL tube and dried at 95 °C for 24 h. After this, the dry biomass was weighed, and its protein content was measured using a Kjeldahl apparatus (JK9870, Jingrui Analysis Instrument Co., Ltd., Jinan, China) while its amino acid composition was analyzed by Zhongyuan Yongxin Technology Co., Ltd. (Beijing, China) using a high-performance liquid chromatography tandem mass spectrometer. To track the evolution of the bacterial community throughout the operation, samples from different stages were sent to the Majorbio Institute (Shanghai, China) for 16S ribosomal RNA (rRNA) amplicon paired-end sequencing and subsequent analysis.

## 3 Results

### 3.1 Microbial electrosynthesis of acetate from CO_2_ in Reactor 1

**Fig. 2:**
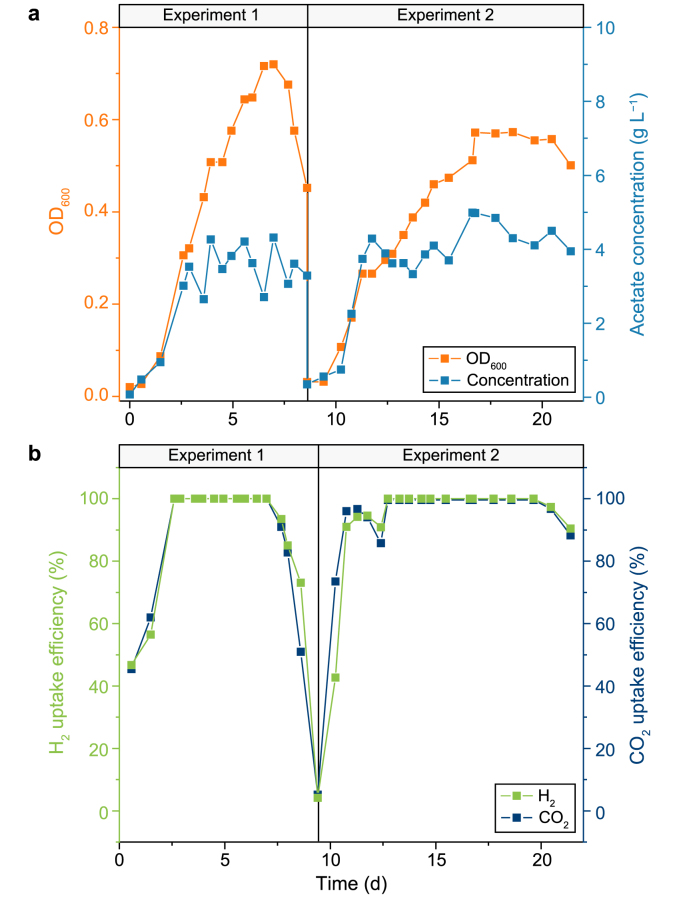
Performance of Reactor 1 with homo-acetogens during the operating period of two experiments. **a**, The optical density at 600 nm (OD_600_) and the concentration of acetate at the electro-bubble column reactor. **b**, Gas uptake efficiency at the electro-bubble column reactor in two experiments.

CO_2_ was reduced to acetate by homo-acetogens in Reactor 1. Then, the catholyte that contained acetate was pumped into Reactor 2, where acetate-utilizing bacteria metabolized acetate, accumulating SCP. Two experiments were conducted to ensure reproducibility. In Experiment 1, acetate production commenced immediately after inoculation of homo-acetogen community (Fig. 2a). Over the first three days, the acetate concentration steadily increased to 3.5 g L^−1^, but it dropped gradually on Day 4 because the culture started to flow. Subsequently, the acetate concentration in Reactor 1 was maintained between 2 and 5 g L^−1^. This continuous MES of acetate from CO_2_ facilitated SCP production by stably providing acetate to acetate-utilizing bacteria. The results of Experiment 2 closely resembled those of Experiment 1. Experiment 1 exhibited a higher bacterial growth rate than Experiment 2, likely due to the former's higher current density (Supplementary Material Table S2). The maximum OD_600_ values recorded for Experiment 1 and Experiment 2 were 0.72 on Day 7 and 0.57 on Day 18.6, respectively. Although the acetate concentrations on Day 7 and Day 18.6 were both 4.3 g L^−1^, the decrease in OD_600_ was quite interesting to observe. Therefore, the limited ATP production in bacteria [14], primarily due to the lack of hydrogen and acetyl coenzyme A (acetyl-CoA) rather than acetate inhibition, may lead to relatively low biomass yield.

The H_2_ and CO_2_ uptake efficiencies of the homo-acetogens were consistent with their growth patterns (Fig. 2b). The gas uptake efficiency approached 100%, except during the start and end periods of each experiment due to the fluctuating environment. However, in Experiment 2, when the current density of Reactor 1 was increased from 3 to 4 A on Day 12.4, the H_2_ uptake efficiency significantly decreased from 96% to 86%. Fortunately, when the current density was reverted to 3 A, the H_2_ uptake efficiency returned to 100%. These results indicate that the unique structure of the electro-bubble column reactor allows for full gas utilization, overcoming the challenge of the limited solubility of H_2_ in water (only 0.79 mM) [15].

### 3.2 Efficient single-cell protein production from acetate by acetate-utilizing bacteria in Reactor 2

**Fig. 3:**
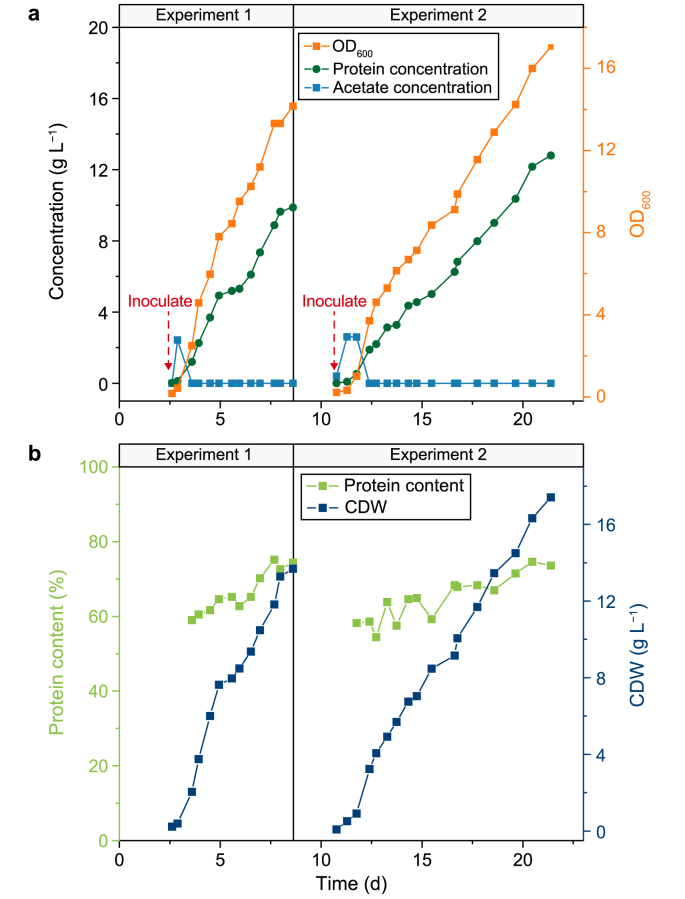
Performance of Reactor 2 with acetate-utilizing bacteria during the operating period of two experiments. **a**, The optical density at 600 nm (OD_600_) and the concentrations of acetate and protein at the stirred tank bioreactor. **b**, The cell dry weight (CDW) of produced single-cell proteins and protein content (wt%) in cell dry weight.

Fig. 3 illustrates the production of SCP during the operation of the biohybrid system. The time profiles of OD_600_, indicative of cell growth, as well as the concentrations of acetate and protein in the fermenter are presented in Fig. 3a. The acetate concentration rapidly increased after inoculation on the first day. Subsequently, acetate-utilizing bacteria consumed acetate more efficiently, decreasing the acetate concentration of the fermentation broth to zero. Then, the clear fermentation broth was pumped back into Reactor 1 at a rate of 7.2 mL min^−1^. In Experiment 1, the OD_600_ reached 14.2 on Day 8.6, corresponding to a CDW of 13.7 g L^−1^. For Experiment 2, the maximal OD_600_ and CDW were recorded as 17.0 and 17.4 g CDW L^−1^, respectively.

A steady increase in the SCP content (SCP/CDW) is shown in Fig. 3b. In Experiment 1, the integrated bioprocess system yielded 9.9 g L^−1^ of SCP, with an SCP content of 74.0% over six days and an average SCP productivity of 1.7 g L^−1^ d^−1^. In Experiment 2, the protein concentration was 12.8 g L^−1^, with a protein rate of 1.2 g L^−1^ d^−1^. Table 1 provides detailed information on the protein production performance. Although the protein production rate is relatively high, the electron-to-protein efficiency is comparatively low in both experiments, at 11.5% and 12.0%, respectively (Supplementary Material Table S4). Consequently, additional measures should be implemented to enhance the electron-to-protein conversion efficiency.

**Table 1:** The results of microbial protein production in this study.

Experiment	Biomass concentration (g CDW L^−1^)	Average yield (g CDW per g ${COD}_{H_{2}}$)	Average volumetric biomass productivity (g CDW L^−1^ d^−1^)	Average volumetric protein productivity (g protein L^−1^ d^−1^)	Protein content
Experiment 1	13.7	0.11	2.2	1.7	74%
Experiment 2	17.4	0.10	1.5	1.2	74%

Abbreviations: CDW, cell dry weight. COD, chemical oxygen demand.

### 3.3 Amino acid profile

**Fig. 4:**
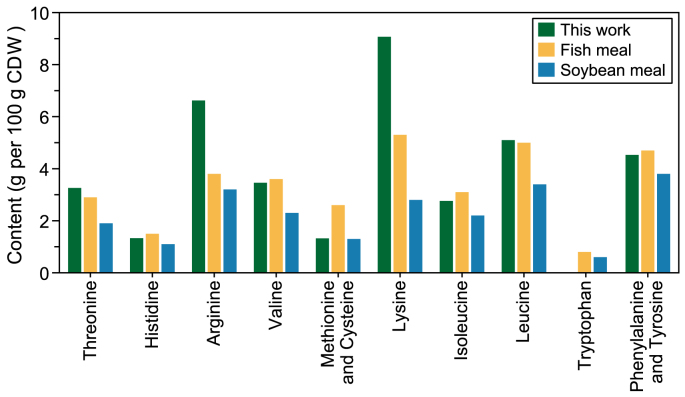
Profile of essential and conditionally essential amino acids in the microbial biomass. Microbial biomass was produced under a stirred tank bioreactor by the *Alcaligenes*-dominated culture (green) (this study), and the amino acid profile was compared with fishmeal (yellow) and soybean meal (blue) as reported by Øverland et al. [16]. CDW: cell dry weight.

The bacterial biomass was harvested on Day 22, after which its essential amino acid composition was analyzed (Fig. 4). Fish and soybean meal were used as reference points for animal and vegetable protein, respectively [16]. Overall, the amino acid profile of the produced SCP closely resembled that of fish meal and surpassed the profile of soybean meal. However, the produced SCP was deficient in sulfur-containing amino acids, such as methionine and cysteine, but had elevated levels of other amino acids. Therefore, the sulfide content of the medium should be increased in the future. Notably, the arginine content of the produced SCP was significantly higher than that of fish meal and soybean meal, reaching 7.1 g per 100 g CDW, which is highly beneficial for shrimp growth [17]. These findings suggest that the produced SCP can be an excellent supplement to fish and soybean meals.

### 3.4 Microbial community stability in the two reactors

**Fig. 5:**
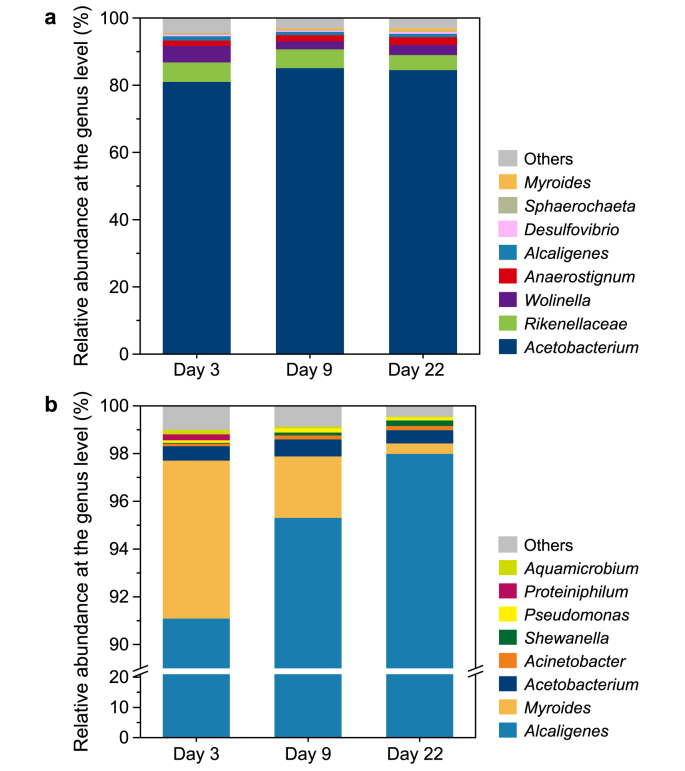
Bacteria community compositions at the genus level of Reactor 1 (**a**) and Reactor 2 (**b**).

DNA samples from the two reactors (at different stages of operation) were sequenced using 16S rRNA Illumina sequencing to assess their microbial community composition. The relative abundance of *Acetobacterium* exceeded 80% in Reactor 1 (Fig. 5a). Fig. 5b shows that almost 96% of the total microbial community in Reactor 2 was composed of a single genus: *Alcaligenes*, which are the most commonly used hydrogen-oxidizing bacteria [11]. This discovery is significant, suggesting that the fermentation operation can be effectively controlled without requiring labor-intensive sterile measures such as autoclaving media and gas filtration. Notably accounting for the overall energy demand for feed production are sterilization of the medium and substrate (3.1–77.0%) and reactor cooling (11.0–54.0%) [18]. The results also show that the microbial communities in Reactor 1 and Reactor 2 are relatively independent and stable.

## 4 Discussion

This study created a hybrid system that effectively produces SCP from CO_2_. The previously reported biohybrid systems typically used an open setup where the medium continuously flowed into and out of the reactor, leading to the wastage of water resources [19,20]. When evaluating the environmental impact of a product, water usage is a critical factor to consider. Effective water management can significantly reduce the negative ecological effects associated with SCP production [18]. For example, in this study, the medium was circulated through an electro-bubble column, where CO_2_ was converted into acetate, and a stirred tank reactor, where acetate-utilizing bacteria converted acetate into SCP. Using circulated media allows for the recovery of nutrients (e.g., mineral salts, biomass lysates, etc.) and inhibits CO_2_ loss in the effluent since the circulated media already contains dissolved CO_2._

In this study, the continuous consumption by the acetate-utilizing bacteria of the acetate produced in Reactor 1 reduced the product inhibition of MES in Reactor 1. Throughout the process, Reactor 1 continuously supplied acetate to Reactor 2 at relatively low concentrations (2–5 g L^−1^), reducing the inhibition of acetate on the acetate-utilizing bacteria. This allowed for rapid startup of Reactor 2 after inoculation, enhancing the SCP production. Moreover, unlike traditional gas fermenters, the electro-bubble reactor generates hydrogen in situ from the cathode. The electrolytic hydrogen gas exists as micro- and nano-bubbles, resulting in the oversaturation of the electrolytic water with hydrogen, which promotes mass transfer. During the stable operating period in this study, the gas uptake efficiency approached ∼100% due to the innovative structure of the electro-bubble column reactor, which addresses mass transfer and safety concerns associated with the direct use of H_2_ under aerobic conditions. More importantly, the biohybrid system could be used not only for SCP (food) production but also for other intracellular product bioprocesses, such as fatty alcohol [21] (material) and ρ-farnesene [22] (fuel) production.

The designed system yielded 12.8 g L^−1^ of SCP from CO_2_, demonstrating an overall productivity of 1.2 g L^−1^ d^−1^ and a high protein content of 74%. Notably, its produced SCP had a higher protein content than fish meal (68%) [16] and soybean meal (48%) [23]. Despite the system's attractive properties, its hollow fiber membranes may be contaminated during the system's long-term operation, although this did not occur in this study. Therefore, the acetate extraction process must be improved in the future. Furthermore, the produced SCP has some limitations regarding its inclusion in human or animal diets. One of its main limitations is its high nucleic acid content, which surpasses other conventional protein sources and may increase uric acid levels in serum, potentially causing kidney stone formation [24]. While 70–80% of nitrogen in microorganisms is present as amino acids, the remaining portion is present as nucleic acids, an important characteristic of fast-growing organisms [25]. In these cases, it is recommended that SCP be used as feed for animals with shorter lifespans, such as salmon, calves, and chickens [26]. Alternatively, various techniques for reducing nucleic acid content can be utilized to make SCP suitable for food applications. Chemical treatments such as sodium chloride and sodium hydroxide, as well as enzymatic treatments such as deoxyribonuclease and ribonuclease, can be applied to biomass to reduce nucleic acid concentrations to below 2% (w/w) [27].

## 5 Conclusion

We have established a novel biohybrid system that couples aerobic *Alcaligenes* and anaerobic homo-acetogens within a medium-recirculating system to produce SCP from CO_2_ and electricity. The results demonstrated that this integrated system with medium recirculation between the two reactors efficiently reduces wastewater generation and alleviates product inhibition. The system not only solves the issues of low acetate values and the difficulty of extracting acetate in MES but also avoids the mass transfer and safety issues associated with using H_2_ under aerobic conditions to produce SCP. The approach developed in this study can be easily extended to generate more valuable products from CO_2_.

## CRediT authorship contribution statement

**Zeyan Pan:** Writing – review & editing, Writing – original draft, Methodology, Investigation, Conceptualization. **Yuhan Guo:** Validation, Investigation. **Weihe Rong:** Resources. **Sheng Wang:** Resources. **Kai Cui:** Validation. **Wenfang Cai:** Conceptualization. **Zhihui Shi:** Resources. **Xiaona Hu:** Validation. **Guokun Wang:** Writing – review & editing, Supervision, Resources. **Kun Guo:** Writing – review & editing, Supervision, Methodology.

## Data availability

Data will be made available on request.

## Declaration of competing interest

The authors declare that they have no known competing financial interests or personal relationships that could have appeared to influence the work reported in this paper.
