# Supplementary material for: Single-cell protein production from CO2 and electricity with a recirculating anaerobic-aerobic bioprocess
Source: Environ Sci Ecotechnol. 2025 Jan 10;24:100525. doi: 10.1016/j.ese.2025.100525 (PMC11787703; doi:10.1016/j.ese.2025.100525)
Supplement: Multimedia component 1 [file mmc1.docx]

**Supporting Information**

**Single-Cell Protein Production from CO_2_ and Electricity with A Recirculating Anaerobic-Aerobic Bioprocess**

Zeyan Pan^a^, Yuhan Guo^a^, Weihe Rong^b^, Sheng Wang^c^, Kai Cui^a^, Wenfang Cai^a^, Zhihui Shi^b^, Xiaona Hu^d^, Guokun Wang^b,*^, Kun Guo^a,*^

*^a^School of Chemical Engineering and Technology, Xi’an Jiaotong University, Xi’an 710049, China*

*^b^Key Laboratory of Engineering Biology for Low-carbon Manufacturing, Tianjin Institute of Industrial Biotechnology, Chinese Academy of Sciences, Tianjin 300308, China*

^c^Shanghai Zelixir Biotech Company Ltd., Shanghai 200030, China

*^d^School of Ecology and Environment, Zhengzhou University, Zhengzhou 450000, PR China.*

***** Corresponding Authors: Guokun Wang, wanggk@tib.cas.cn;

Kun Guo, [kun.guo@xjtu.edu.cn](mailto:kun.guo@xjtu.edu.cn)


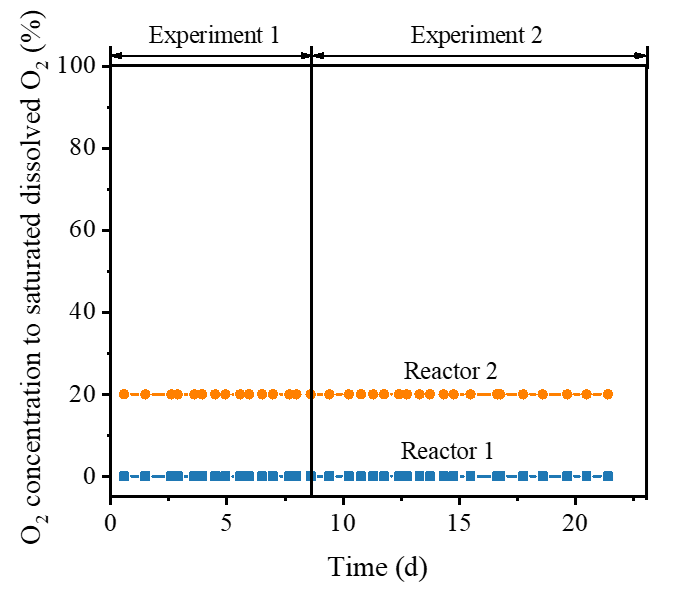


**Figure S1**. DO level in two reactors.


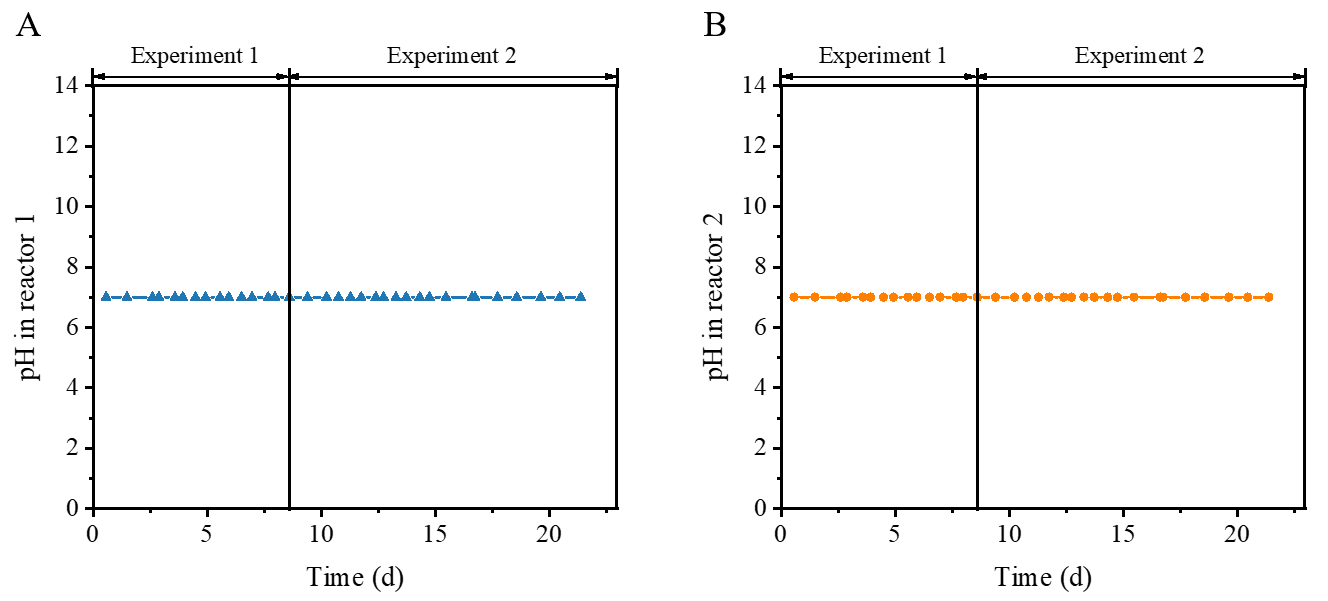


**Figure S2**. pH in reactor 1 (A) and reactor 2 (B).


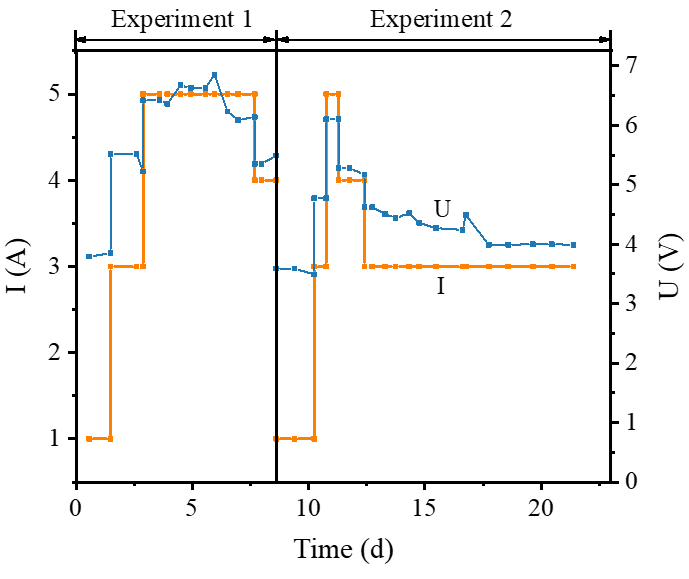


**Figure S3.** The current and corresponding voltage applied to the electro-bubble column reactor.

**Table S1.** Composition of trace element solution and vitamin solution

| **Trace element solution** | | **Vitamin solution** | |
| --- | --- | --- | --- |
| Chemical | g/L | Chemical | g/L |
| KI | 0.18 | Biotin | 0.002 |
| H_3_BO_3_ | 0.15 | Folic acid | 0.002 |
| CuSO_4_·5H_2_O | 0.03 | Vitamin B12 | 0.0001 |
| FeCl_3_·6H_2_O | 1.5 | Riboflavin | 0.05 |
| MnCl_2_·4H_2_O | 0.12 | Thiamine | 0.05 |
| CoCl_2_·6H_2_O | 0.15 | Nicotinic acid | 0.05 |
| ZnSO_4_·7H_2_O | 0.12 | Pantothenic acid | 0.05 |
| Na_2_MoO_4_·2H_2_O | 0.06 | Pyridoxine-HCl | 0.01 |
| EDTA (acid form) | 10 | p-Aminobenzoic acid | 0.05 |
| NaOH | Adjust pH to 7 | Thioctic acid | 0.05 |
| NiCl_2_·6H_2_O | 0.023 | \|  \|  \| \| --- \| --- \| |  |

**Table S2.** Operation conditions of the anaerobic electro-bubble column reactor

| Experiment 1 | | | Experiment 2 | | | |
| --- | --- | --- | --- | --- | --- | --- |
| Operation duration  (h) | Galvanostatic current  (A) | CO_2_ flow rate (mL/min) | Operation duration  (h) | Galvanostatic current  (A) | | CO_2_ flow rate (mL/min) |
| 0-36 | 1 | 4 | 206-245 | | 1 | 4 |
| 36-69 | 3 | 12 | 245-259 | | 3 | 12 |
| 69-184 | 5 | 20 | 259-271 | | 5 | 20 |
| 184-206 | 4 | 16 | 271-298 | | 4 | 16 |
| - | - | - | 298-513 | | 3 | 12 |

**Table S3.** Essential amino acid content (g/100 g CDW) of biomass in this work, fish meal and soybean meal

|  | Biomass in  this work | Fish meal | Soybean meal |
| --- | --- | --- | --- |
| Histidine | 1.5 | 1.5 | 1.1 |
| Threonine | 2.9 | 2.9 | 1.9 |
| Arginine | 7.1 | 3.8 | 3.2 |
| Valine | 3.3 | 3.6 | 2.3 |
| Methionine and Cysteine | 0.2 | 2.6 | 1.3 |
| Lysine | 5.5 | 5.3 | 2.8 |
| Isoleucine | 1.8 | 3.1 | 2.2 |
| Leucine | 3.1 | 5 | 3.4 |
| Tryptophan | 0 | 0.8 | 0.6 |
| Phenylalanine and Tyrosine | 4.3 | 4.7 | 3.8 |

Table S4. Electron balance of two experiments in this study

|  | Electron balance | | | |
| --- | --- | --- | --- | --- |
|  | H_2_ | Acetate | SCP | Others |
| Experiment 1 | 7.2% | 8.8% | 11.5% | 72.5% |
| Experiment 2 | 4.9% | 8.8% | 12.0% | 74.3% |

**S1. Calculations**

The production rate (g product/L/d) and yield (g product/g substrate) parameters were calculated to evaluate the overall performance of reactors. The biomass production rate (Eq. (1)) was calculated as the ratio of total suspended solids production and the reactor working volume (L_R_) over the entire bioconversion period (n), where TSS_n_ and TSS_0_ represent the final and initial mass of dry biomass, respectively. Similarly, the protein production rate (Eq. (2)) was calculated as the ratio of total protein production and the reactor working volume over the bioconversion period, where SCP_n_ and SCP_0_ represent the final and initial mass of biomass protein, respectively. The biomass yield was calculated as the ratio of total suspended solids production and the total amount of COD available through the feeding of gases given by Eq. (3). The gas uptake efficiency (Eq. (4)) was calculated as the ratio of the utilized gas volume and the total inflow gas volume, where Gas_in_ and Gas_out_ represent the total inflow gas volume and the total outflow gas volume, respectively.

$Biomass production rate=\frac{TSS_{n}-{TSS}_{0}}{L_{R}\times n}$ [g biomass / L reactor working volume /d] (1)

$Protein production rate=\frac{SCP_{n}-{SCP}_{0}}{L_{R}\times n}$ [g protein / L reactor working volume /d] (2)

$Biomass yield_{Y_{H_{2}}}=\left( \frac{gCDW}{{gH}_{2}-COD} \right)=\frac{\Delta CDW \left( g/L \right) \times Liquid volume (L)}{\Delta H_{2}\left( mol \right) \times16 (g COD/mol H_{2})}$ (3)

$Gas uptake efficiency=\frac{{Gas}_{in}-{Gas}_{out}}{{Gas}_{in}}\times100\%$ [%] (4)

The calculation process of electron balance was detailed as follows:

In experiment 1, the total electrons applied:

$$n_{{total-e}^{-}}=\frac{Q}{F}=\frac{\Sigma It}{F}=\frac{2882400 C}{96485 C/mol}=29.874 mol$$

The electrons to H_2_:

$$n_{e^{-}\to H_{2}}=\frac{V_{H_{2-out}}}{V_{m}}\times2=\frac{26.812 L}{24.862 L/mol}\times2=2.157 mol$$

The electrons to acetate:

$$n_{e^{-}\to{CH}_{3}COOH}=\frac{m_{{CH}_{3}COOH}}{M_{{CH}_{3}COOH}}\times8=\frac{19.74 g}{60 g/mol}\times8=2.632 mol$$

The electrons to SCP:

$$n_{e^{-}\to SCP}=n_{e^{-}\to{COD}_{s}}\times4=\frac{m_{{COD}_{s}}}{M_{O_{2}}}\times4=\frac{18.35 g/L \times1.5 L}{32 g/mol}\times4=3.441 mol$$

The electron efficiency of electron to H_2_:

$${EE}_{e^{-}\to H_{2}}=\frac{n_{e^{-}\to H_{2}}}{n_{{total-e}^{-}}}=\frac{2.157}{29.874}\times100\%=7.2\%$$

The electron efficiency of electron to CH_3_COOH:

$${EE}_{e^{-}\to{CH}_{3}COOH}=\frac{n_{e^{-}\to{CH}_{3}COOH}}{n_{{total-e}^{-}}}=\frac{2.632}{29.874}\times100\%=8.8\%$$

The electron efficiency of electron to SCP:

$${EE}_{e^{-}\to SCP}=\frac{n_{e^{-}\to SCP}}{n_{{total-e}^{-}}}=\frac{3.441}{29.874}\times100\%=11.5\%$$

The electron efficiency of electron to others:

$${EE}_{e^{-}\to others}=1-{EE}_{e^{-}\to H_{2}}-{EE}_{e^{-}\to{CH}_{3}COOH}-{EE}_{e^{-}\to SCP}=72.5\%$$

Similarly, the electron balance for experiment 2 could also be calculated.
